# Relationship between long-term outcomes and optimal time interval in patients with bilateral synchronous multiple primary lung cancers: a multi-institutional cohort study

**DOI:** 10.1080/07853890.2025.2590200

**Published:** 2025-11-21

**Authors:** Wei Guo, Bolun Zhou, Qilin Huai, Ying Ji, Ying Chen, Huayu He, Liankui Han, Bangsheng Li, Junzheng Zhou, Fengwei Tan, Qi Xue, Shugeng Gao, Jie He

**Affiliations:** ^a^ Department of Thoracic Surgery, National Cancer Center/National Clinical Research Center for Cancer/Cancer Hospital, Chinese Academy of Medical Sciences and Peking Union Medical College, Beijing, People’s Republic of China; ^b^ Key Laboratory of Minimally Invasive Therapy Research for Lung Cancer, Chinese Academy of Medical Sciences, Beijing, People’s Republic of China; ^c^ Department of Thoracic Surgery, Beijing Chao-Yang Hospital, Capital Medical University, Beijing, People’s Republic of China; ^d^ Department of Thoracic Surgery, Yunnan Cancer Hospital, Kunming, People’s Republic of China; ^e^ Department of Thoracic Surgery, Peking University Third Hospital, Beijing, People’s Republic of China; ^f^ Department of Thoracic Surgery, Guizhou Provincial People’s Hospital, Guizhou, People’s Republic of China; ^g^ Department of Thoracic Surgery, Anyang Cancer Hospital, Anyang, People’s Republic of China

**Keywords:** Multiple primary lung cancer, surgery, synchronous, overall survival

## Abstract

**Background:**

With the advancement of diagnostic technology and changes in living environment, the incidence of synchronous multiple primary lung cancer (sMPLC) is increasing. This study aims to investigate the impact of surgical timing on clinical outcomes.

**Methods:**

This multi-institutional cohort study retrospectively enrolled 901 patients with bilateral sMPLC who underwent two surgeries from January 2017 to December 2022. Three different subgroups were determined based on the time interval between the 1^st^ surgery and the 2^nd^ surgery: Group-I (time interval ≤ 90 days), Group-II (90 days < time interval ≤ 180 days) and Group-III (time interval > 180 days).

**Results:**

For the entire cohort, the median follow-up time from the second surgery was 49.2 months. The clinical outcomes of patients in Group-II were more favourable than those in Group-I and Group-III (log-rank, OS, *p* = 0.0037; DFS, *p* < 0.0001). According to multivariable Cox regression analyses, patients in Group-I (HR, 1.704; 95% CI, 0.828–3.51; *p* = 0.148) and Group-III (HR, 5.369; 95% CI, 2.664–10.819; *p* < 0.001) may be associated with poor prognosis compared with those in Group-II. In addition, patients in Group-III had a high incidence of postoperative complications, and significantly reduced lung function was identified among patients in Group-I.

**Conclusions:**

For patients with bilateral sMPLC, our research identified a potential correlation between the interval of subsequent surgeries and patient prognosis. We suggest that an interval of 90 to 180 days between surgeries may be the most beneficial.

## Introduction

With the commonplace utilization of high-resolution computed tomography (CT), detection of lung cancer has increased significantly in recent years [1]. Lung cancer has emerged as one of the leading causes of cancer-specific mortality globally [2]. In certain instances, individuals are found to have multiple pulmonary nodules, and the prevalence of such cases is steadily rising [3]. Multiple primary lung cancer (MPLC) may be diagnosed simultaneously or at different times, categorized as synchronous MPLC (sMPLC) or metachronous MPLC (mMPLC) [4]. A major diagnostic challenge in MPLC cases is distinguishing whether co-existing nodules represent intrapulmonary metastases or primary lung cancer, which could help determine the following therapeutic strategies [5]. Diagnosing multifocal lesions preoperatively can sometimes be challenging based on clinical and radiological features, while histological and genetic variations among co-existing lesions are considered reliable indicators for sMPLC [6].

Multiple surgical approaches are employed to remove the primary lesion and accompanying ipsilateral pulmonary nodules in sMPLC [7]. Patients with sMPLC exhibit acceptable survival outcomes after surgery, especially for those with early-stage disease [8]. Nevertheless, the situation becomes complicated in cases of sMPLC when lesions are present in bilateral lobes. On one hand, the challenge of performing a second surgery increases for many patients, and optimal surgical strategy for bilateral sMPLC is lacking. This includes considerations such as determining the optimal time interval for re-resection and the order of surgeries for each side at the initial consultation, which may affect the long-term prognosis of patients [9]. Moreover, some patients may be intolerant to the second surgery, especially the elderly and those experiencing severe adverse events after the initial surgery. Some novel treatment strategies for those patients are being investigated for managing residual lesions, such as radiation therapy and immunotherapy [10].

In the present study, we have assessed the feasibility and safety of surgery, compared the subsequent surgeries and evaluated the long-term prognosis of individuals with bilateral sMPLC. To our knowledge, no study has proposed a reliable and effective strategy for bilateral sMPLC. This multi-institution analysis aimed to demonstrate the optimal surgical treatment paradigms for bilateral sMPLC, offering novel insights into the management of bilateral sMPLC.

## Materials and methods

### Patient cohort

From January 2017 to December 2022, a total of 901 patients with bilateral sMPLC who underwent two surgeries were enrolled from six high-volume academic hospitals in China. The inclusion criteria were as follows: patients with [1] primary carcinoma of the lung [2]; more than one primary lung cancer confirmed by the pathology report [3]; lesions in bilateral sites [4]; different pathological origin of lesions based on pathology [5]; two surgical resections. Patients meeting the following criteria were excluded: patients with [1] two surgical resections for lesions of only one side [2]; distant metastasis or contralateral mediastinal lymph node metastasis [3]; preoperative chemotherapy, radiotherapy, targeted therapy or immunotherapy [4]; incomplete clinical information.

Preoperative evaluation included physical examination, bronchoscopy, CT scans of the neck, chest and abdomen, pulmonary function, whole-body radionuclide bone scan, and brain magnetic resonance imaging. Mediastinoscopy, bronchial ultrasound-guided transbronchial puncture, or positron emission tomography were used to evaluate metastasis in mediastinal lymph nodes. For postoperative follow-up, we recommended patients to have chest computed tomography (CT) scans every 6 months for the 1^st^ year after surgery and annually thereafter for as long as possible. OS was defined as the time from the date of the second surgery to death from any cause. DFS was defined as the time from the second surgery to recurrence or death.

### Surgical approach

The surgical strategy for patients with bilateral sMPLC is determined by the surgeon based on preoperative evaluation. Generally, among patients with bilateral sMPLC, the primary lesion with the largest diameter, a higher percentage of solid component, closer to the hilum and pleura, and with a higher risk of lymph node spread should be treated first [11]. However, in some cases, surgical prioritization was given to lesions characterized by smaller diameters, a lower percentage of solid components, and minimal lymph node invasion. This approach was considered an unconventional surgical strategy, and the optimal sequencing of the two surgeries was investigated in this study.

### Pathological evaluation

For lesions sharing the same pathological type, the determination of whether they were metastases can be made by referring to the patient’s genetic test report. In the absence of genetic test results, experienced pathologists assessed the pathological subtype to ultimately determine whether the lesions qualify as MPLC. Determining the primary lesion was further based on preoperative imaging assessing the nodal size and solid component, as well as on the degree of differentiation and risk factors outlined in the postoperative pathology report, in cases where pathological staging was the same.

### Subgroups determined by the time interval

We calculated the time interval between the date of the first surgery and the date of the second surgery. For most patients receiving the surgery, post-surgical follow-up visits are commonly recommended at 3 and 6 months after surgery. Herein, we stratified all patients into three subgroups: Group-I (time interval ≤ 90 days), Group-II (90 days < time interval ≤ 180 days) and Group-III (time interval > 180 days). We aimed to compare the difference in survival outcomes among patients of three different subgroups and found the best timing for the second surgery in patients with bilateral sMPLC.

### Statistical analysis

Continuous variables were presented as mean (SD) or median (IQR), while categorical variables were reported as counts with percentages. We performed the Mann–Whitney U-test and t-test to assess variations between continuous variables with non-normal and normal distributions, respectively. We used Fisher’s exact test or Chi-square test to assess variations between categorical variables. The log-rank test was used to compare DFS and OS calculated from the date of the 2^nd^ surgery in the cohort. The univariate and multivariable Cox proportional hazards regression models were used to evaluate the associations between different clinical characteristics and prognosis. Variables exhibiting a univariate P-value below 0.05 were selected for inclusion in multivariable regression models. Statistical analyses were performed using R software (version 4.0.4), with a 2-sided P-value < 0.05 considered statistically significant.

### Declaration of Helsinki statement

The present study has been performed in accordance with the principles stated in the Declaration of Helsinki.

## Results

### General characteristics of patients

Of 901 patients with bilateral sMPLC, 299 patients (33.2%) were male and 602 patients (66.8%) were female. For the largest lesion, the median diameter was 1.80 cm (interquartile range, 1.20–2.50 cm), and the consolidation tumor ratio (CTR) of the major lesion was more than 0.5 in 521 patients (57.8%). For the entire cohort, the median follow-up time from the second surgery was 49.2 months and the median time interval was 122 days. The clinicopathologic characteristics of patients in three subgroups are presented in Table 1.

**Table 1.** Clinicopathologic characteristics of patients with bilateral synchronous multiple primary lung cancer.

Characteristics	Overall	Group-I	Group-II	Group-III	*P* Value
	901	312	271	318	
Gender (%)					
Male	299 (33.2)	103 (33.0)	87 (32.1)	109 (34.3)	0.853
Female	602 (66.8)	209 (67.0)	184 (67.9)	209 (65.7)	
Age at the 1st surgery (median [IQR])	59.00 [53.00, 65.00]	59.00 [53.00, 64.00]	59.00 [52.50, 66.00]	58.00 [52.00, 64.00]	0.437
BMI (median [IQR])	23.50 [21.60, 25.40]	23.45 [21.50, 25.42]	23.40 [21.50, 25.40]	23.60 [21.70, 25.17]	0.87
Smoking history (%)					
Never	678 (75.2)	239 (76.6)	213 (78.6)	226 (71.1)	0.085
Yes/Ever	223 (24.8)	73 (23.4)	58 (21.4)	92 (28.9)	
Family history (%)					
Never	672 (74.6)	238 (76.3)	198 (73.1)	236 (74.2)	0.661
Yes/Ever	229 (25.4)	74 (23.7)	73 (26.9)	82 (25.8)	
Location of the largest tumor (%)					
Left lower lobe	116 (12.9)	37 (11.9)	38 (14.0)	41 (12.9)	0.947
Left upper lobe	299 (33.2)	110 (35.3)	89 (32.8)	100 (31.4)	
Right lower lobe	135 (15.0)	48 (15.4)	39 (14.4)	48 (15.1)	
Right middle lobe	65 (7.2)	25 (8.0)	19 (7.0)	21 (6.6)	
Right upper lobe	286 (31.7)	92 (29.5)	86 (31.7)	108 (34.0)	
CTR of the largest tumor (%)					
≤0.5	380 (42.2)	127 (40.7)	109 (40.2)	144 (45.3)	0.375
>0.5	521 (57.8)	185 (59.3)	162 (59.8)	174 (54.7)	
Number of lesions (%)					
2	436 (48.4)	156 (50.0)	121 (44.6)	159 (50.0)	0.424
3	244 (27.1)	92 (29.5)	77 (28.4)	75 (23.6)	
4	122 (13.5)	33 (10.6)	43 (15.9)	46 (14.5)	
5	51 (5.7)	18 (5.8)	14 (5.2)	19 (6.0)	
>5	48 (5.3)	13 (4.2)	16 (5.9)	19 (6.0)	
Diameter of the largest tumor (median [IQR])	1.80 [1.20, 2.50]	1.80 [1.30, 2.62]	1.70 [1.20, 2.50]	1.80 [1.20, 2.50]	0.225
The sequence of surgery determined by tumor size (%)					
Unconventional	314 (34.9)	119 (38.1)	97 (35.8)	98 (30.8)	0.144
Conventional	587 (65.1)	193 (61.9)	174 (64.2)	220 (69.2)	
The sequence of surgery determined by the number of lesions (%)					
Unconventional	710 (78.8)	240 (76.9)	214 (79.0)	256 (80.5)	0.545
Conventional	191 (21.2)	72 (23.1)	57 (21.0)	62 (19.5)	
The number of deaths (%)	33 (3.7)	13 (4.2)	4 (1.5)	16 (5.0)	0.061
The number of recurrences (%)	81 (9.0)	30 (9.6)	11 (4.1)	40 (12.6)	0.001
Follow-up time (median [IQR])	49.20 [29.47, 65.50]	55.77 [35.27, 73.00]	53.23 [33.13, 70.37]	33.56 [26.04, 50.57]	<0.001

### Features of two consecutive surgeries

To evaluate differences between the two surgeries, we compared the features of two consecutive surgeries among patients across three subgroups (Table S1). In the 1st surgery patients in Group-III had more lesions resected than those in Group-I and Group-II, whereas no significant difference was observed among patients in the 2^nd^ surgery. In the 1^st^ surgery, the majority of patients underwent lobectomy, particularly those in Group-II (50.6%) and Group-III (57.2%). In contrast, a relatively higher proportion of patients in Group-I underwent wedge resection (28.2%). In the 2^nd^ surgery, the results were quite different. The rate of wedge resection was relatively higher in Group-II (42.8%) and Group-III (43.7%), whereas the majority of patients in Group-I underwent lobectomy (37.2%).

### Pathological characteristics of resected lesions

The pathological type of the major lesion tended to be similar among the three subgroups in the 1^st^ surgery, whereas the difference was observed in the 2^nd^ surgery (Table S2). In the 1^st^ surgery, the major lesion of more than 70% of patients was adenocarcinoma. In the 2^nd^ surgery, the proportion of patients with adenocarcinoma as the major lesion was 71.8% of patients in Group-I, which was higher than those in Group-II (63.8%) and Group-III (58.8%). The proportion of solid/micropapillary subtype, spread through air spaces (STAS) and vessel carcinoma embolus (VCE) was similar among three subgroups in both surgeries.

### Differences in pre-operative pulmonary function tests among three subgroups

We assessed the pre-operative pulmonary function tests before two surgeries. We also compared differences of two pulmonary function tests to evaluate the effect of the initial surgery on the pulmonary function among patients in the three subgroups (Table S3). The second pulmonary function tests were conducted 2–4 weeks before the second surgery. According to our results, the pulmonary function declined for the majority of patients after the 1^st^ surgery, particularly for patients in Group-I. In addition, the decline of pulmonary function was most obvious for patients in Group-I, whereas it was least obvious for patients in Group-III.

### Survival time of patients with different intervals between two surgeries

**Figure 1.**
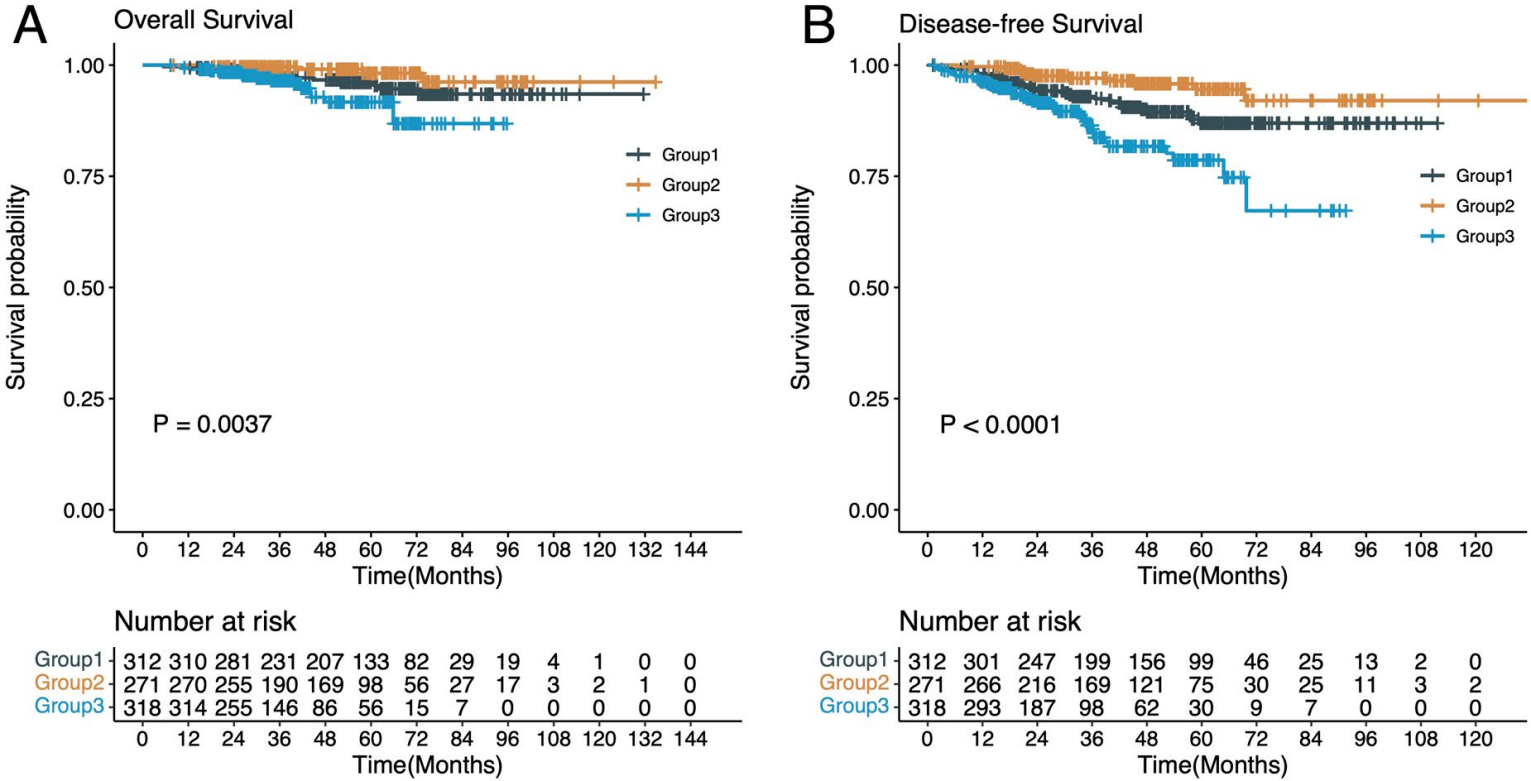
Overall survival (OS) and disease-free survival (DFS) of patients with bilateral synchronous multiple primary lung cancer (sMPLC) in different subgroups. A. OS from the date of the second surgery for patients with sMPLC (*p* = 0.0037). B. DFS from the date of the second surgery for patients with sMPLC (*p* < 0.0001).

For the entire cohort, the median follow-up time from the second surgery was 49.2 months. The patients in Group-II (90 days < time interval ≤ 180 days) were associated with more favorable OS and DFS compared with patients in Group-I (time interval ≤ 90 days) and Group-III (time interval > 180 days) after the 2^nd^ surgery (Figure 1(A–B), OS, *p* = 0.0037; DFS, *p* < 0.0001). According to univariate and multivariable Cox regression analyses, patients in Group-I (HR, 1.704; 95% CI, 0.828–3.51; *p* = 0.148) and Group-III (HR, 5.369; 95% CI, 2.664–10.819; *p* < 0.001) were correlated with poorer prognosis than Group-II (Table 2). The univariate and multivariable Cox regression analyses for OS showed similar trends (Table S4).

**Table 2.** Univariate and multivariable cox regression analyses for disease-free survival.

	Univariate		Multivariable	
Characteristics	HR (95% CI)	*P* Value	HR (95% CI)	*P* Value
Interval subgroup				
Group-I	2.311 (1.158–4.611)	0.018	1.704 (0.828–3.51)	0.148
Group-II	1 [Reference]		1 [Reference]	
Group-III	4.605 (2.352–9.016)	<0.001	5.369 (2.664–10.819)	<0.001
Gender				
Male	1 [Reference]		1 [Reference]	
Female	0.339 (0.218–0.529)	<0.001	0.424 (0.225–0.801)	0.008
Age at the 1st surgery	1.024 (0.998–1.05)	0.069	1.015 (0.987–1.043)	0.293
Smoking history				
Never	1 [Reference]		1 [Reference]	
Yes/Ever	2.61 (1.686–4.043)	<0.001	0.834 (0.444–1.568)	0.574
Diameter of the biggest lesion	1.812 (1.55–2.119)	<0.001	1.447 (1.1–1.905)	0.008
CTR of the major lesion				
≤0.5	1 [Reference]		1 [Reference]	
>0.5	5.285 (2.641–10.578)	<0.001	3.275 (1.562–6.863)	0.002
Number of lesion (1st surgery)	0.803 (0.562–1.148)	0.229		
Number of lesion (2nd surgery)	0.669 (0.459–0.975)	0.036	0.751 (0.501–1.125)	0.164
Solid or micropapillary (1st surgery)				
No	1 [Reference]		1 [Reference]	
Yes	2.609 (1.67–4.074)	<0.001	1.832 (1.112–3.019)	0.017
Solid or micropapillary (2nd surgery)				
No	1 [Reference]		1 [Reference]	
Yes	2.001 (1.171–3.42)	0.011	0.701 (0.374–1.314)	0.268
Number of resected lymph nodes (1st surgery)	1.02 (0.992–1.049)	0.159		
Number of resected lymph nodes (2nd surgery)	1.046 (1.019–1.074)	0.001	1.003 (0.971–1.037)	0.851
Pathological stage (1st surgery)				
0	1 [Reference]		1 [Reference]	
IA	1.624 (0.393–6.711)	0.503	0.707 (0.161–3.102)	0.646
IB	2.809 (0.644–12.254)	0.169	0.498 (0.1–2.474)	0.394
IIA	6.46 (1.077–38.734)	0.041	0.172 (0.02–1.454)	0.106
IIB	5.587 (1.183–26.373)	0.03	0.701 (0.118–4.172)	0.696
IIIA	12.484 (2.649–58.832)	0.001	2.035 (0.357–11.608)	0.424
IIIB	9.82 (0.888–108.573)	0.062	0.405 (0.023–7.079)	0.536
Pathological stage (2nd surgery)				
0	1 [Reference]		1 [Reference]	
IA	1.289 (0.513–3.237)	0.589	0.773 (0.298–2.009)	0.598
IB	4.342 (1.545–12.202)	0.005	1.315 (0.361–4.793)	0.678
IIA	8.246 (1.598–42.555)	0.012	2.353 (0.389–14.224)	0.351
IIB	20.602 (6.281–67.578)	<0.001	1.304 (0.261–6.523)	0.747
IIIA	9.253 (2.674–32.016)	<0.001	1.737 (0.37–8.161)	0.484
VPI (1st surgery)				
No	1 [Reference]			
Yes	0.965 (0.521–1.788)	0.91		
VPI (2nd surgery)				
No	1 [Reference]		1 [Reference]	
Yes	3.83 (2.186–6.71)	<0.001	1.401 (0.597–3.289)	0.438
VCE (1st surgery)				
No	1 [Reference]		1 [Reference]	
Yes	2.651 (1.402–5.013)	0.003	1.069 (0.491–2.329)	0.866
VCE (2nd surgery)				
No	1 [Reference]		1 [Reference]	
Yes	5.969 (3.297–10.806)	<0.001	3.171 (1.38–7.285)	0.007

CTR, consolidation tumor ratio; VPI, visceral pleural invasion; VCE, vessel carcinoma embolus.

### Comparison of 30-day postoperative complications after two subsequent surgeries

Compared to those after the 1^st^ surgery, the overall rates of complications were relatively lower after the 2^nd^ surgery (Table 3). The most prevalent complication was sleep disturbance in the 1^st^ (21.2%) and the 2^nd^ surgeries (13.2%). In the 1^st^ surgery, the rates of post-operative haemorrhage (0.3% and 0.4% vs 3.8%), pneumonia (0.3% and 2.2% vs 3.5%), subcutaneous emphysema (1.6% and 2.2% vs 5.3%) and thromboembolism (1.3% and 0.4% vs 6.0%) were significantly higher for patients in Group-III. In contrast, the rates of constipation (10.9% and 8.5% vs 15.1%), subcutaneous emphysema (2.2% and 1.8% vs 6.9%) and thromboembolism (1.9% and 1.5% vs 4.7%) were also higher for patients in Group-III after the 2^nd^ surgery.

**Table 3.** Summary of 30-day postoperative complications.

Characteristics	Complications after the 1st surgery				Complications after the 2nd surgery			
	Group-I	Group-II	Group-III	*P* Value	Group-I	Group-II	Group-III	*P* Value
Sleep disturbance (%)	68 (21.8)	53 (19.6)	70 (22.0)	0.73	48 (15.4)	31 (11.4)	40 (12.6)	0.343
Nausea and vomiting (%)	28 (9.0)	24 (8.9)	38 (11.9)	0.349	22 (7.1)	19 (7.0)	37 (11.6)	0.064
Anuresis (%)	9 (2.9)	7 (2.6)	4 (1.3)	0.34	8 (2.6)	5 (1.8)	6 (1.9)	0.786
Constipation (%)	48 (15.4)	30 (11.1)	53 (16.7)	0.138	34 (10.9)	23 (8.5)	48 (15.1)	0.039
Persistent pulmonary leakage (%)	14 (4.5)	11 (4.1)	16 (5.0)	0.851	7 (2.2)	12 (4.4)	11 (3.5)	0.337
Atelectasis (%)	4 (1.3)	4 (1.5)	17 (5.3)	0.002	4 (1.3)	5 (1.8)	10 (3.1)	0.249
Post-operative hemorrhage (%)	1 (0.3)	1 (0.4)	12 (3.8)	<0.001	2 (0.6)	2 (0.7)	7 (2.2)	0.14
Fever (%)	11 (3.5)	18 (6.6)	28 (8.8)	0.024	10 (3.2)	12 (4.4)	15 (4.7)	0.602
Supraventricular tachyarrhythmia (%)	2 (0.6)	6 (2.2)	8 (2.5)	0.165	6 (1.9)	5 (1.8)	5 (1.6)	0.941
Pneumonia (%)	1 (0.3)	6 (2.2)	11 (3.5)	0.018	3 (1.0)	4 (1.5)	7 (2.2)	0.45
Subcutaneous emphysema (%)	5 (1.6)	6 (2.2)	17 (5.3)	0.015	7 (2.2)	5 (1.8)	22 (6.9)	0.001
Chylothorax (%)	1 (0.3)	5 (1.8)	5 (1.6)	0.192	1 (0.3)	2 (0.7)	0 (0.0)	0.301
Thromboembolism (%)	4 (1.3)	1 (0.4)	19 (6.0)	<0.001	6 (1.9)	4 (1.5)	15 (4.7)	0.031
Infection (%)	4 (1.3)	3 (1.1)	9 (2.8)	0.206	4 (1.3)	3 (1.1)	4 (1.3)	0.979
Respiratory failure (%)	0 (0.0)	1 (0.4)	3 (0.9)	0.2	1 (0.3)	1 (0.4)	4 (1.3)	0.271

### The survival benefits brought by conventional surgical strategies

Generally, the 1^st^ surgery tended to be conducted on the side with larger or more lesions, which was the conventional surgical strategy for bilateral sMPLC. Most surgeons tended to conduct the 1^st^ surgery on the side with larger lesions, while performing the 1^st^ operation on the side with more lesions was not typically a priority (Table 1). According to our results, no significant difference was observed in OS (*p* = 0.78) and DFS (*p* = 0.84) between the conventional and unconventional subgroups determined by the tumor size, suggesting that the conventional surgical strategy may not improve the prognosis of patients with bilateral sMPLC (Figure S1A-B). Furthermore, no benefit was observed in OS (*p* = 0.96) and DFS (*p* = 0.46) for those who received the 1^st^ surgery on the side with more lesions (Figure S1C-D).

## Discussion

Previous studies have reported that patients with sMPLC accounted for 2% of patients with lung cancer, with more than two-thirds being ipsilateral lesions [12]. With increased awareness of early lung cancer screening, an increasing number of patients are being diagnosed with sMPLC. Bilateral sMPLC, a rare type of lung cancer, presents significant challenges in determining whether surgery is appropriate and in selecting the most effective surgical strategies for bilateral lesions.

It is crucial to distinguish between sMPLC and advanced lung cancer, as they have significantly different prognoses and treatment modalities. We only enrolled patients with bilateral sMPLC with 91.1% of patients being pathological stage 0 or stage I, which was similar to the pathological characteristics of MPLC patients reported in previous publications [13]. Surgery is unquestionably the preferred option for treating patients with MPLC, including bilateral sMPLC [14]. The clinical outcomes tended to be similar for sMPLC patients who underwent surgery compared to similarly staged patients with solitary primary lung cancer [15]. However, the surgical strategies for bilateral sMPLC, especially regarding when and how to perform two subsequent surgeries, remains controversial. Our findings revealed that surgeons tended to prioritize the largest lesion for the 1^st^ surgery, whereas the number of lesions on each side may not be an important factor for them in determining the surgical sequence. Although resecting smaller and more accessible lesions in the 1^st^ surgery may be controversial, some surgeons followed this strategy to reduce the time and risk for patients (unconventional surgical strategy) [16]. We did not identify significant differences in OS and DFS between patients in conventional and unconventional subgroups, suggesting that the traditional way of determining the surgical sequence may be inappropriate.

Ethun et al. found that time interval may be a risk factor for patients with incidental gallbladder cancer who received reoperation, and the optimal time interval between the two surgeries could bring significant survival benefits [9]. The recommended time point for the 2^nd^ surgery was from 90 to 180 days after the 1^st^ surgery. According to the survival analysis, patients in Group-III (time interval > 180 days) exhibited significantly worse prognosis than those in Group-I (time interval ≤ 90 days) and Group-II, and time interval may be an important contributor. Other reasons may also lead to the long-time interval and poor prognosis of patients in Group-III. For instance, patients in Group-III had slightly more postoperative complications after the first and second surgeries, which may be one of the reasons for the longer time interval and worse prognosis. And some other non-clinical factors may also lead to a delay in the Group-III, such as patient preference and institutional schedule. Previous studies have revealed the clinical characteristics of MPLC, and implied that the survival probability of sMPLC and metachronous MPLC were similar [13]. However, Shintani et al. have only mentioned that the time interval was less than 2 years between the two surgeries for patients with sMPLC and not further assessed the potential effect of time interval on the prognosis of patients [13]. Our study has proposed that the time interval between the 1^st^ and 2^nd^ surgeries may affect the prognosis for patients with bilateral sMPLC. In addition, we have found that pulmonary function declined for the majority of patients after the 1^st^ surgery. However, the decline seemed to be associated with the time interval between the two surgeries and was not related to the prognosis of patients.

There are some limitations in the present study. First, due to the retrospective nature of this study, an inherent selection bias exists by only enrolling patients receiving the 2^nd^ surgery, while this is prevalent in studies analyzing the effect of surgery timing on patient prognosis. Second, since we did not know why patients underwent their second operation at a particular time, selection bias may make these results difficult to interpret. Third, although the median follow-up time from the date of the 2^nd^ surgery is nearly 5 years, longer follow-up is needed for the cohort.

## Conclusions

Specifically, an interval of 90 to 180 days between surgeries is suggested as optimal, offering valuable implications for informing surgical strategies in these patients with bilateral sMPLC.

## Supplementary Material

TableS1.xlsx

FigureS1.jpg

Table S4.xlsx

TableS3.xlsx

Supplemental Materials legend.docx

TableS2.xlsx

## Data Availability

The data that supporting the findings of this study can be obtained from the corresponding author upon reasonable request.
