# Supplementary figures and images for: Relationship between long-term outcomes and optimal time interval in patients with bilateral synchronous multiple primary lung cancers: a multi-institutional cohort study
Source: Ann Med. 2025 Nov 21;57(1):2590200. doi: 10.1080/07853890.2025.2590200 (PMC12642896; doi:10.1080/07853890.2025.2590200)

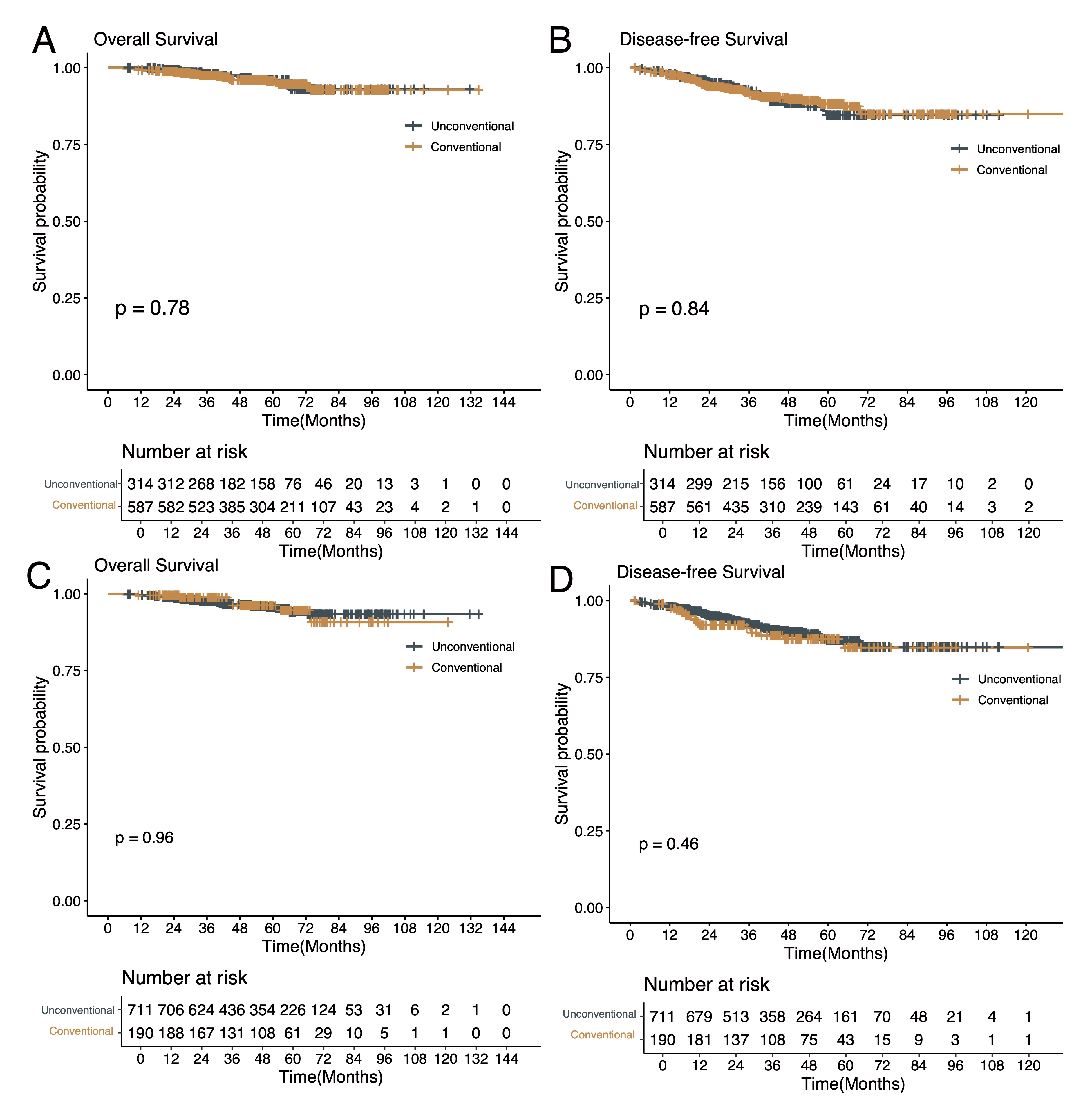

Supplement: FigureS1.jpg [file IANN_A_2590200_SM4242.jpg]
