# Supplementary material for: Relationship between long-term outcomes and optimal time interval in patients with bilateral synchronous multiple primary lung cancers: a multi-institutional cohort study
Source: Ann Med. 2025 Nov 21;57(1):2590200. doi: 10.1080/07853890.2025.2590200 (PMC12642896; doi:10.1080/07853890.2025.2590200)
Supplement: Supplemental Materials legend.docx [file IANN_A_2590200_SM4239.docx]

Supplemental Materials

**Figure S1. Overall survival (OS) and disease-free survival (DFS) of patients with bilateral synchronous multiple primary lung cancer (sMPLC) in different subgroups.** A-B. Kaplan-Meier curves comparing OS and DFS between two subgroups stratified by the sequence of surgery. The conventional approach involves first removing the lesions of the side with larger tumors, followed by the removal of lesions of the side with smaller tumors. C-D. Kaplan-Meier curves comparing OS and DFS between two subgroups stratified by the sequence of surgery. The conventional approach involves first removing the lesions of the side with more lesions, followed by the removal of lesions of the side with fewer lesions.

**Table S1.** Characteristics of the two consecutive surgeries.

**Table S2.** Pathological characteristics of patients with bilingual synchronous multiple primary lung cancer.

**Table S3.** The results of pre-operative lung function tests.

**Table S4.** Univariate and multivariable Cox regression analyses for overall survival.
